# Tetrandrine alleviates inflammation and neuron apoptosis in experimental traumatic brain injury by regulating the IRE1α/JNK/CHOP signal pathway

**DOI:** 10.1002/brb3.2786

**Published:** 2022-11-14

**Authors:** Huan Liu, Shiqing He, Chong Li, Jianpeng Wang, Qin Zou, Yongshi Liao, Rui Chen

**Affiliations:** ^1^ Department of Cardiology, Affiliated Nanhua Hospital University of South China Hengyang China; ^2^ Department of Neurosurgery, Affiliated Nanhua Hospital University of South China Hengyang China

**Keywords:** apoptosis, CHOP, JNK, TBI, Tetrandrine

## Abstract

**Aim:**

The aim of this study was to investigate the therapeutic roles of Tetrandrine (TET) on traumatic brain injury (TBI) and the underlying mechanism.

**Method:**

Traumatic injury model of hippocampal neurons and TBI mouse model were established to evaluate the therapeutic effects. The expression of neuron‐specific enolase (NSE), Caspase 3, and Caspase 12 was detected by immunofluorescence. The expression of TNF‐α, NF‐κB, TRAF1, ERS markers (GADD34 and p‐PERK), IRE1α, CHOP, JNK, and p‐JNK were evaluated by western blot. Flow cytometry was used to determine the apoptosis of neurons. Brain injury was assessed by Garcia score, cerebral water content, and Evan blue extravasation test. Hematoxylin and eosin staining was used to determine the morphological changes of hippocampal tissue. Apoptosis was assessed by TUNEL staining.

**Result:**

In traumatic injury model of hippocampal neurons, TET downregulated NSE, TNF‐α, NF‐κB, TRAF1, GADD34, p‐PERK, IRE1α, CHOP, and p‐JNK expression. TET reduced Caspase 3 and Caspase 12 cleavage. Apoptosis rate was inhibited by the introduction of TET. TET improved the Garcia neural score, decreased the cerebral water content and Evans blue extravasation, and reduced NSE, TNF‐α, NF‐κB, TRAF1, IRE1α, CHOP, and p‐JNK expression in mice with TBI, which was significantly reversed by Anisomycin, a JNK selective activator.

**Conclusion:**

TET alleviated inflammation and neuron apoptosis in experimental TBI by regulating the IRE1α/JNK/CHOP signal pathway.

## Graphical abstract

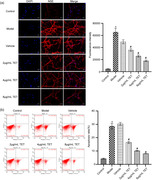

## 1 INTRODUCTION

Traumatic brain injury (TBI) is mainly caused by blunt mechanical forces and has been regarded as one of the main global significant public health and socioeconomic issues (Van Horn et al., 2017). The morbidity and mortality of TBI are rising year by year, which makes TBI the leading cause for death and disability of young adults (Dang et al., 2017). The pathological process of focal or diffuse brain injury induced by TBI includes the axon shear, cell edema, vascular injury, and neuronal death (Esterov & Greenwald, 2017; Gardner et al., 2017; Russo & McGavern, 2016). Series of pathological progresses, such as inflammation post primary brain injury (Corrigan et al.2016), endoplasmic reticulum stress (ERS) inhibition (Lucke‐Wold et al., 2016), the formation of oxygen radicals (Angeloni et al., 2015; Y. Wang et al., 2016), calcium release and dysfunction of mitochondria (Rao et al., 2015), will contribute to the secondary brain injury or even neuronal death. Compared with other mechanisms, the elapsed time to cause cell death by inflammation is more durable, which provides broader therapeutic time window for drug interference to avoid secondary damages.

The activation of microglia and the recruitment of surrounding neutrophils can be induced by the early stage of TBI, followed by the infiltration of macrophages derived from lymphocytes and monocytes. Meanwhile, pro‐inflammatory factors, such as interleukins (IL‐1, IL‐12, IL‐18), interferon‐γ (IFN‐γ), and tumor necrosis factor α (TNF‐α), compete with the anti‐inflammatory factors, such as interleukins (IL‐4, IL‐10, IL‐13) and interferon‐α (IFN‐γ), to promote or inhibit the neuro inflammatory reactions, respectively. The directed migration and activation of immune cells can be induced by the chemotactic factors, such as CCL5. In this way, cell debris is cleared by the inflammatory reactions, which is beneficial to the recovery of TBI. However, the induced apoptosis of neurons and progressive neurodegeneration will deteriorate the condition of TBI, which contributes to the focal and diffuse brain injury (Lozano et al., 2015; Morioka et al., 2019; Paiva et al.2015). Therefore, it is of great significance to alleviate inflammation post TBI and the apoptosis of neurons to avoid amplified damage caused by secondary brain injury.

Tetrandrine (TET) is one of the active ingredients isolated from Tsuzura, which is a Chinese traditional medicine. TET is broadly reported to be involved in such biological functions as anti‐inflammation, analgesia, anti‐lipid peroxidation, cell protective, anti‐fibrosis, and anti‐tumor effects (He et al., 2011; He et al., 2011; Wu et al., 2011). TET exerts anti‐inflammatory effects by inhibiting the activation and proliferation of T cells, macrophages, and neutrophils or by inhibiting the calcium channel to decrease the concentration of Ca^2+^ within the inflammatory cells and the activity of Ca^2+^‐CaM, which finally suppresses the release of pro‐inflammatory factors (J. Chen et al., 2010). In the present study, the anti‐inflammatory effects and neuron protective effects of TET will be investigated to claim its potential therapeutic effects on TBI.

## 2 MATERIALS AND METHODS

### 2.1 Isolation of mouse hippocampal neurons

The hippocampal neurons were obtained from CD‐1 mice aged 6‐‐8 weeks. The mice were covered by C‐section under Nembutal anesthesia. Hippocampal tissue was aseptically separated on an ultra‐clean table. After the tissue was cut up with scissors, it was loaded into a 15 ml centrifuge tube. Papain with a final concentration of 5 ml 2 mg/ml was added and mixed. The tissue was digested at 37°C for 10 min, and the centrifuge tube was reversed every 5 min. DMEM/F12 medium containing fetal bovine serum was added to terminate digestion. After centrifugation at 1000 rpm for 10 min, the supernatant was discarded, and the centrifugation was repeated once. Note that 5 ml DMEM/F12 (including DNASEI) medium was added, and pasteur pipet was gently blown 30–40 times to make cell suspension, which was passed through a 200‐mesh filter. After centrifugation at 1000 r/min for 5–8 min, plating medium (98% neurobasal media, 2% B27 supplement, 0.5 mM glutamine solution, 25 μM glutamate, green streptomycin, 10% heat inactivated horse serum 1 mM HEPES) was used to resuspend cells. The resuspended cells were placed on slides coated with Poly‐D‐lysine (0.1 mg/ml) in a culture dish with a density of 4 × 10^5^ cells/ml and cultured in an incubator at 37°C. On the second day, all the medium was replaced with serum‐free culture medium (98% Neurobasal Media, 2% B27 Supplement, 0.5 mM Glutamine solution, 1 mm Hepes). Afterward, the liquid was changed every 2–3 days by half amount.

We declare that all animal experiments involved in this study were authorized by the ethical committee of Affiliated Nanhua Hospital, University of South China and carried out according to the guidelines for care and use of laboratory animals and the principles of laboratory animal care and protection. The protocol number is ANH2019122, and the title of the study was “TET alleviates inflammation and neuron apoptosis in experimental TBI by regulating the IRE1α/JNK/CHOP signal pathway.”

### 2.2 Traumatic injury model of hippocampal neurons and intervention

Each petri dish was manually scratched with a sterile plastic pipette tip in a 9 × 9 square grid to construct a hippocampal neuron cell trauma model. After scratching, the culture was washed with phosphate buffered saline (PBS) to remove cell debris. Live cells were counted by calcein staining. The scratch caused about 38% of the cells in the culture dish to be removed.

To investigate the roles of TET on hippocampal neurons after traumatic injury, the cells were divided into five groups, including control group, model group, vehicle group, 2 μg/ml TET group, 4 μg/ml TET group, and 8 μg/ml TET group. Cells in the control group were left untreated. The model group was established by the traumatic injury model according to the above methods. Traumatic hippocampal neurons were treated with 2 μg/ml, 4 μg/ml, and 8 μg/ml TET for 24 h, respectively, as 2 μg/ml TET group, 4 μg/ml TET group, and 8 μg/ml TET group. Traumatic hippocampal neurons in the vehicle group were treated with DMSO (TET vehicle). To determine whether the effect of TET on traumatic hippocampal neurons was related to the IRE1α/JNK/CHOP signal pathway, traumatic hippocampal neurons in the Anisomycin group and the TET + Anisomycin group were treated with 10 nM Anisomycin for 24 h (Shen et al., 2019), and traumatic hippocampal neurons in the TET + Anisomycin group were treated with TET for 24 h.

### 2.3 Western blot assay

Protein was extracted from cells using protein extraction kit (Beyotime, China). Approximately 40 μg of protein was extracted on a 12% sodium dodecyl sulfate‐polyacrylamide gel (SDS‐PAGE). By electrophoresis, the gel was transferred to a polyvinylidene fluoride membrane (Millipore). The membrane was sealed with 5% skimmed milk powder at room temperature for 1 h. The membrane was incubated with the primary antibody overnight, including TNF‐α (Abcam, 1:1000, UK), NF‐κB (Abcam, 1:1000, UK), TRAF1 (Abcam, 1:1000, UK), GADD34 (10449‐1‐AP, 1:1000, USA), PERK (Abcam, 1:1000, UK), p‐PERK (CST, 1:1000, USA), IRE1α (27528‐1‐AP, 1:1000, USA), CHOP (27528‐1‐AP, 1:1000, USA), JNK (ab179461, 1:1000, UK), p‐JNK (ab124956, 1:6000, UK), and internal reference β‐actin (66009‐1‐Ig, 1:1000, USA). The membrane was incubated with secondary antibody HRP Goat‐Anti‐Mouse IgG (SA00001‐1, 1:5000, USA) or HRP Goat‐Anti‐Rabbit IgG (SA00001‐2, 1:6000, USA) at 37°C for 90 min. The membrane was incubated with the ECL reagents (Beyotime) and exposed to detect protein expression.

### 2.4 Immunofluorescence

Cell slides were fixed with 4% paraformaldehyde for 30 min. The cell slides were added with 0.3% triton‐100 and permeable at 37°C for 30 min. The hippocampus of each animal was collected and fixed with 4% paraformaldehyde for 24 h.

The hippocampus was dehydrated with gradient ethanol, and xylene was transparent. The hippocampus was embedded in paraffin wax. The 4‐μm thick sections were prepared by paraffin slicer (YD‐315, Yidi, China). The slice was baked at 60°C for about 8 h. The slice was dewaxed and rehydrated with xylene and gradient ethanol. The antigens were repaired by microwave heating.

Cells and tissues were sealed with 5% bovine serum albumin at 37°C for 60 min. The cell slides and tissues were incubated with primary anti‐NSE (66150‐1‐Ig, mouse, 1:50, Proteintech), anti‐Caspase 12 (55238‐1‐AP, Rabbit, 1:50, Proteintech), and anti‐Caspase 3 (66470‐2‐Ig, mouse, 1:50, Proteintech) antibody overnight at 4°C. After three washes with PBS, cells and tissues were incubated with secondary antibody coralite 488‐conjugated affinipure Goat Anti‐Mouse IgG (H+L) (SA00013‐1, 1:200, Proteintech), coralite 594‐conjugated affinipure Goat Anti‐Mouse IgG (H+L) (SA00013‐3, 1:200, Proteintech), and coralite 488‐conjugated affinipure Goat Anti‐Rabbit IgG (H+L) (SA00013‐2, 1:200, Proteintech) for an additional 30 min at room temperature. The DAPI was added to dye the nuclear for 5 min, and 50% glycerinum was used to block the medium. Stained tissues were photographed under a fluorescence microscope (Olympus, Tokyo, Japan).

### 2.5 Flow cytometry for testing the apoptosis

Note that 10 μl of fluorescently labeled Annexin V and 5 μl of propidium iodide (PI) was added to each tube of cells. After incubating at room temperature for 10 min in the dark, the number of apoptotic cells was detected by flow cytometry.

### 2.6 The establishment of TBI model in CD‐1 mouse and intervention

Adult CD‐1 male mice (6–8 weeks) were purchased from the Animal Center of the Chinese Academy of Sciences (Shanghai, China). Animals had free access to food and water under a 12 h light/dark cycle. The mice were anesthetized with isoflurane. The brain was induced stereotactically. The head hair of the mice was shaved and disinfected with iodophor. An incision was made in the middle of the scalp to expose the skull. A dental drill was used to remove the bone flap (5 mm from the left upper temporal lobe) to expose the cortex. The dura mater remained intact. The controlled cortical impact was carried out by a controlled impactor device. The speed was 4 m/s and the depth was 1 mm. The impact duration was 150 ms. Through the above operations, we constructed the TBI model. No controlled cortical impact was performed in the sham group, and the rest of the operations were the same as those in the model group.

Five groups (*n* = 5) were divided in the experiment, which were sham, TBI model, vehicle, 30 mg/kg TET (Jia et al., 2019), and 30 mg/kg TET combined with Anisomycin (TET + Anisomycin). Normal saline was chosen as the vehicle to dissolve TET and Anisomycin in which pH was adjusted to 7.2. Mice in the TET group and the TET + Anisomycin group were administered by oral administration 30 mg/kg TET for 7 days. Meanwhile, mice in the TET + Anisomycin group were administered by intra‐hippocampal injections at a concentration of 100 μg/μl in a volume of 0.5 μl/side (Pena et al., 2014). The sham and TBI model groups were given equal dose of saline orally and intra‐hippocampal injection. At the end of the experiment, the mice were euthanized, and samples were collected for subsequent testing.

### 2.7 Behavioral test

The Garcia test includes seven items, including spontaneous movement, axial sensation, tactile proprioception, limb symmetry, rollover, forelimb extension, and climbing. The maximum score is 21 points. A score of 0 represents the worst performance, and a score of 3 represents the best performance. The sum of the scores of the seven items is the Garcia test score.

### 2.8 Measurement of cerebral water content

After the behavioral test, the brain of each mouse was separated. After removing blood and cerebrospinal fluid with filter paper, the wet weight was measured using an analytical balance. The samples were dried in an oven at 100°C for 24 h. The dry weight was measured using an analytical balance. Brain water content = (wet weight − dry weight)/wet weight × 100%.

### 2.9 Evans blue extravasation assay

The Evans blue (EB) extravasation method was used to assess the integrity of the blood‐brain barrier (BBB). Mice were injected with 0.25 ml of 2% EB dye via the tail vein. After 2 h, the mice were injected with saline and were anesthetized to death. Brain tissue was collected and observed EB extravasation.

### 2.10 Hematoxylin and eosin staining

The hippocampus was fixed by 4% paraformaldehyde for more than 24 h. After gradient alcohol and xylene, the hippocampus was embedded in paraffin. A paraffin microtome (YD‐315, Yidi) was used to prepare 2 μm thick sections. The slices were baked at 60°C for about 8 h. The sections were deparaffinized and rehydrated through xylene and gradient ethanol. The cytoplasm was stained with eosin. The nucleus was stained with hematoxylin. The samples were dehydrated with gradient ethanol. Xylene was used to improve transparency. The neutral adhesive was used to seal the samples. The pictures were taken under an inverted microscope (Olympus).

### 2.11 Enzyme‐linked immunosorbent assay

According to the instruction of the manufacturer, enzyme‐linked immunosorbent assay (ELISA) kits (TNF‐α, CSB‐E11987r; NF‐κB, CSB‐E13148r; CUSABIO, China; TRAF1, E02T0901, BLUE GENE, China) were used to detect the concentration of TNF‐α, NF‐κB, and TRAF1 in the hippocampal homogenate of each mouse. The sample concentration was calculated according to the standard curve.

### 2.12 TdT‐mediated dUTP nick‐end labeling

The hippocampus was fixed in 4% paraformaldehyde for more than 24 h. After gradient alcohol and xylene, the hippocampus was embedded in paraffin. A paraffin microtome (YD‐315, Yidi) prepared 2 μm thick sections. The slices were baked at 60°C for about 8 h. The sections were deparaffinized and rehydrated through xylene and gradient ethanol. The test was carried out strictly according to the experimental procedures of the TdT‐mediated dUTP nick‐end labeling (TUNEL) kit (KGA704, KeyGEN BioTech, China). After sealing the slides with buffer glycerin, the results were observed under an optical microscope (BA210T, MOTIC, Singapore). Note that 3–5 fields of view for each specimen were randomly selected. The Image‐Pro‐Plus software was used to evaluate the proportion of cell apoptosis in each group (apoptosis rate = the number of apoptotic nuclei/the total number of nuclei under the field of view × 100%).

### 2.13 Statistical analysis

All data were expressed as mean ± standard deviation. All experiments were repeated three times independently. Student's *t*‐test was used to compare the differences between the two groups. One‐way analysis of variance was used to compare the differences between the three groups using the Graph Pad Prism 8.0 statistical software. *p* < .05 was considered statistically significant.

## 3 RESULTS

### 3.1 TET alleviated the damage and apoptosis of traumatized hippocampal neurons in vitro

**FIGURE 1:**
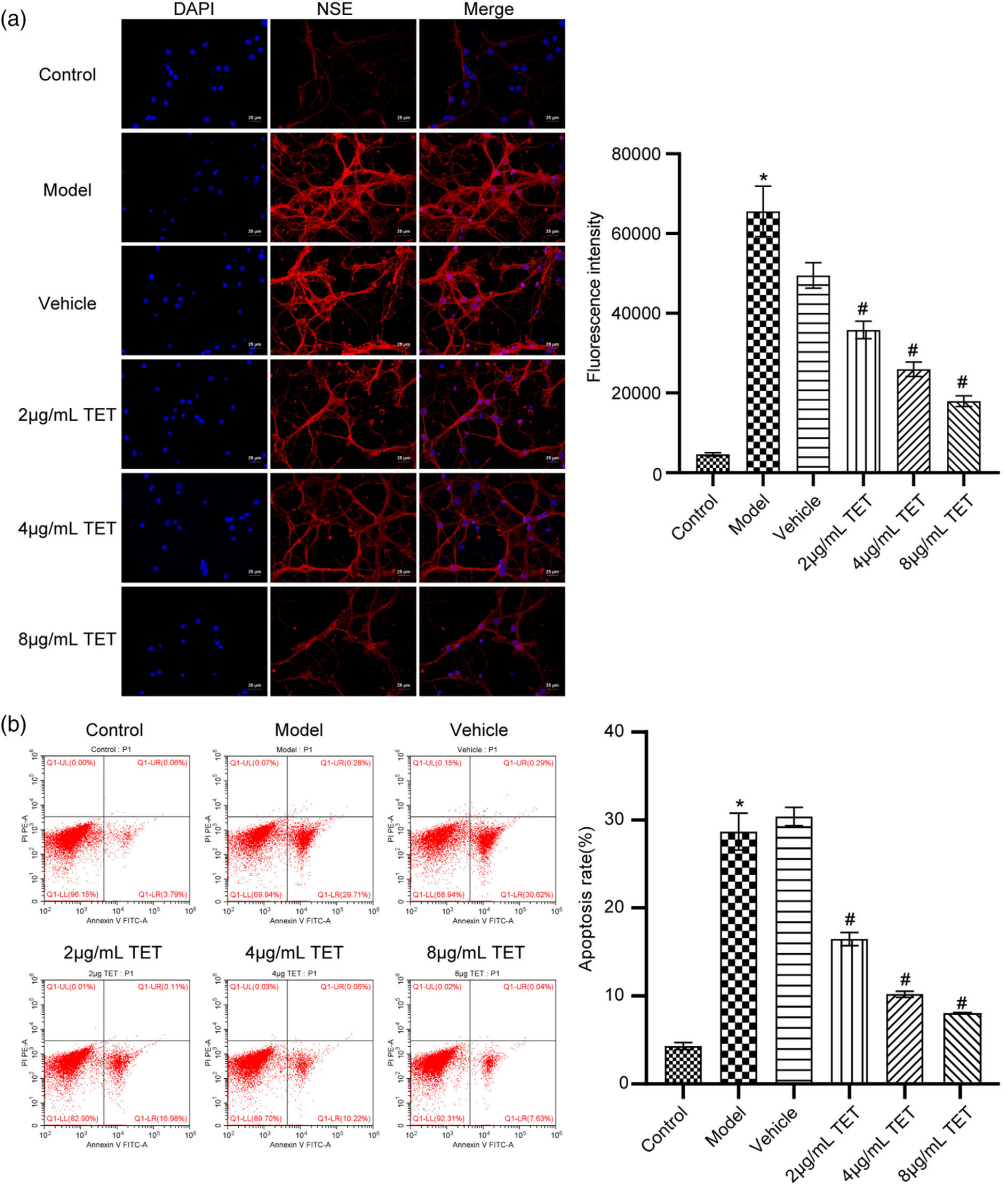
Tetrandrine (TET) alleviated the traumatic injury in vitro. (a) Neuron‐specific enolase (NSE) expression was evaluated by immunofluorescence assay. (b) Flow cytometry was used to determine the apoptosis rate of neuron cells. *p < .05 versus control group, #*p* < .05 versus vehicle group

To claim the effects of TET on hippocampal neurons with traumatic injury, we treated the cells with different concentrations of TET. As shown in Figure 1a, compared with control, NSE was highly expressed in the model groups. The fluorescence intensity decreased greatly as the concentration of TET increased from 0 to 8 μg/ml, which showed a dose‐dependent manner. The apoptosis results are shown in Figure 1b. TET reduced the apoptosis of the traumatized cells in a dose‐dependent manner.

### 3.2 TET attenuated the inflammation of traumatized hippocampal neurons and inhibited the IRE1α/JNK/CHOP signaling pathway in vitro

**FIGURE 2:**
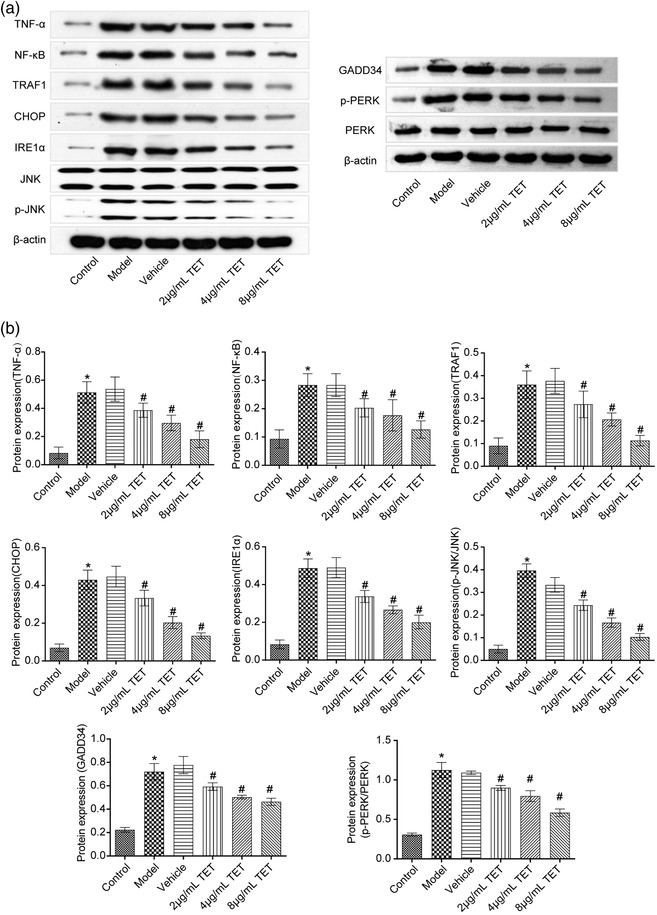
TNF‐α, NF‐κB, TRAF1, GADD34, PERK, p‐PERK, IRE1α, CHOP, JNK, and p‐JNK expression in the neuron cells was detected by western blot. *p < .05 versus control group, #*p* < .05 versus vehicle group

To investigate the effects of TET on inflammation and IRE1α/JNK/Chop signaling pathway in traumatized hippocampal neurons, we performed a western blot assay. As shown in Figure 2, TNF‐α, NF‐κB, TRAF1, GADD34, p‐PERK, IRE1α, CHOP, and p‐JNK were significantly upregulated by traumatic injury modeling on mouse hippocampal neurons, which was downregulated by TET in a dose‐dependent manner. There was no significant difference in PERK and JNK expression among all groups. Now that TET alleviated the traumatic injury in a dose‐dependent manner, 8 μg/ml TET was used for the subsequent experiments.

### 3.3 Anisomycin reversed the effects of TET on traumatic injury in vitro

**FIGURE 3:**
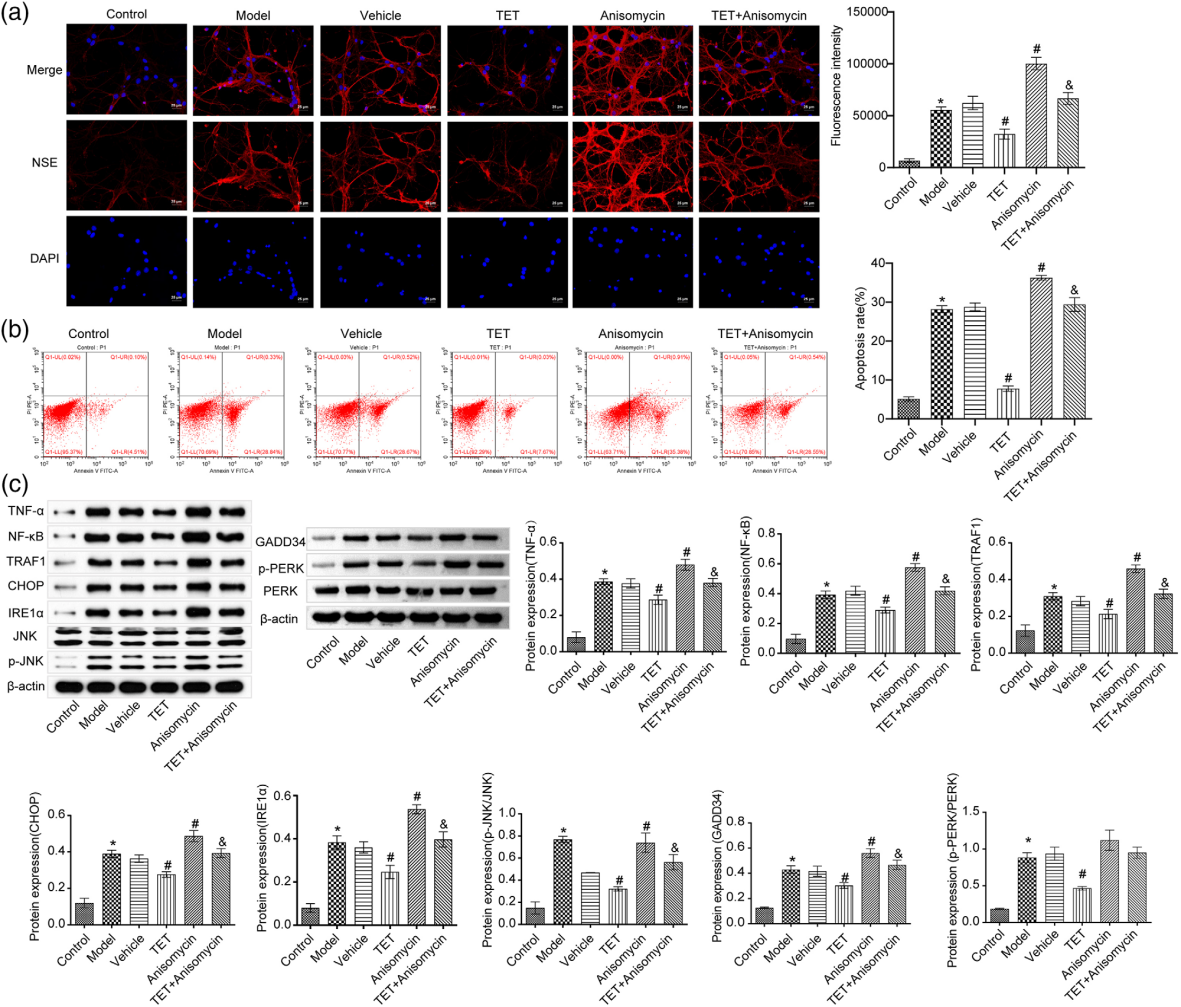
Anisomycin reversed the effects of tetrandrine (TET) on traumatic injury in vitro. (a) Neuron‐specific enolase (NSE) expression was evaluated by immunofluorescence assay. (b) Flow cytometry was used to determine the apoptosis rate of neuron cells. (c) TNF‐α, NF‐κB, TRAF1, GADD34, PERK, p‐PERK, IRE1α, CHOP, JNK, and p‐JNK expression in the neuron cells was detected by western blot. *p < .05 versus control group, #*p* < .05 versus vehicle group, &*p* < .05 versus TET group

To explore whether the effects of TET on traumatic injury was related to the IRE1α/JNK/CHOP signal pathway, Anisomycin, a JNK selective activator, was used to be administered combined with TET. As shown in Figure 3a, the fluorescence intensity was decreased in TET group compared with the vehicle group, which was promoted by the combination of TET and Anisomycin. The combination of TET and Anisomycin partially neutralized the inhibitory effect of TET on apoptosis of traumatized hippocampal neurons (Figure 3b). Figure 3c shows that TET downregulated the high expression level of TNF‐α, NF‐κB, TRAF1, GADD34, p‐PERK, IRE1α, CHOP, and p‐JNK in the traumatic injury hippocampal neurons, which was promoted by the combination of TET and Anisomycin. There was no statistical significance in PERK and JNK expression among all groups. These data indicated that the inhibitory effects of TET on the inflammation and the IRE1α/JNK/CHOP signal pathway were significantly reversed by Anisomycin. In vitro results showed that TET alleviated neuronal injury through the IRE1α/JNK/CHOP signaling pathway.

### 3.4 Anisomycin reversed the effects of TET on Garcia neuro score, cerebral water content, EB extravasation, histomorphological changes, and NSE expression in TBI mice

**FIGURE 4:**
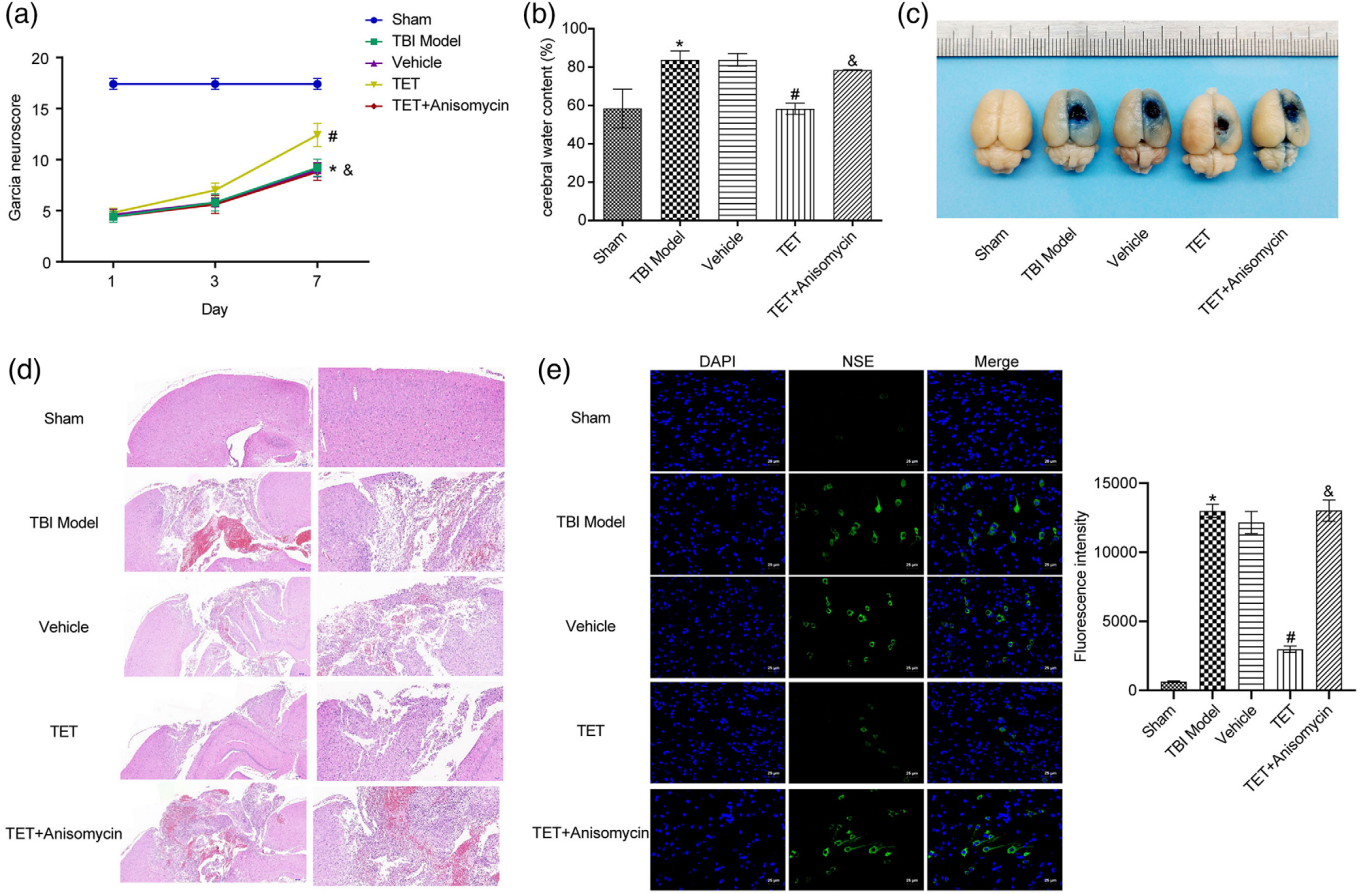
The therapeutic effects of tetrandrine (TET) on traumatic brain injury (TBI) mouse model were reversed by Anisomycin. (a) The Garcia neuro scores of each animal was evaluated. (b) The cerebral water content of each brain was calculated. (c) The degree of brain injury of each brain was determined by Evans blue (EB) extravasation assay. (d) The morphological changes of each hippocampus were evaluated by hematoxylin and eosin (H&E) staining. (e) Neuron‐specific enolase (NSE) expression was evaluated by immunofluorescence assay. *p < .05 versus control group, #*p* < .05 versus vehicle group, &*p* < .05 versus TET group

To investigate the therapeutic effects of TET on TBI in animal model and verify the possible mechanism, TET and Anisomycin were combined to treat TBI mice. As shown in Figure 4a, with the passage of time, the Garcia neuro scores remained unchanged in the sham group and increased in the other four groups. The Garcia neuro score in the TET group was promoted since day 3 compared with the TBI model group, and was decreased by the coadministration of Anisomycin. Figure 4b shows the results of cerebral water content of each tested mouse. Compared with the sham group, higher percentage of water was detected in the TBI model mice, and the cerebral water content was significantly promoted by administering TET, which was reversed by the coadministration of Anisomycin. The EB assay (Figure 4c) showed that deep and rich EB dyes were observed in the brain from TBI model mice compared with the sham group, and fading EB dyes were observed in the brain from TBI mice treated with TET. Interestingly, similar degree of EB dyes was observed in the TET + Anisomycin group. Hematoxylin and eosin (H&E) staining was used to evaluate the morphological changes of each hippocampal tissue, the results of which were shown in Figure 4d. The intact cortex and hippocampus were observed in the sham group. Significant cortical and hippocampal lesions were observed in the model group. However, the degree of cortical and hippocampal lesions was decreased in the TET group, which was reversed by TET combined with Anisomycin. Then, the distribution of NSE protein in the hippocampus was further detected by immunofluorescence. As shown in Figure 4e, green fluorescence represented NSE protein. Intensive fluorescence was observed in the hippocampal neurons isolated from TBI model mice compared with the sham group, which was suppressed by the treatment of TET. However, the fluorescence intensity by coadministering with Anisomycin was promoted to the extent of the TBI model group.

### 3.5 Anisomycin reversed the effects of TET on apoptosis, inflammation‐related and IRE1α/JNK/CHOP signaling pathway‐related proteins in TBI mice

**FIGURE 5:**
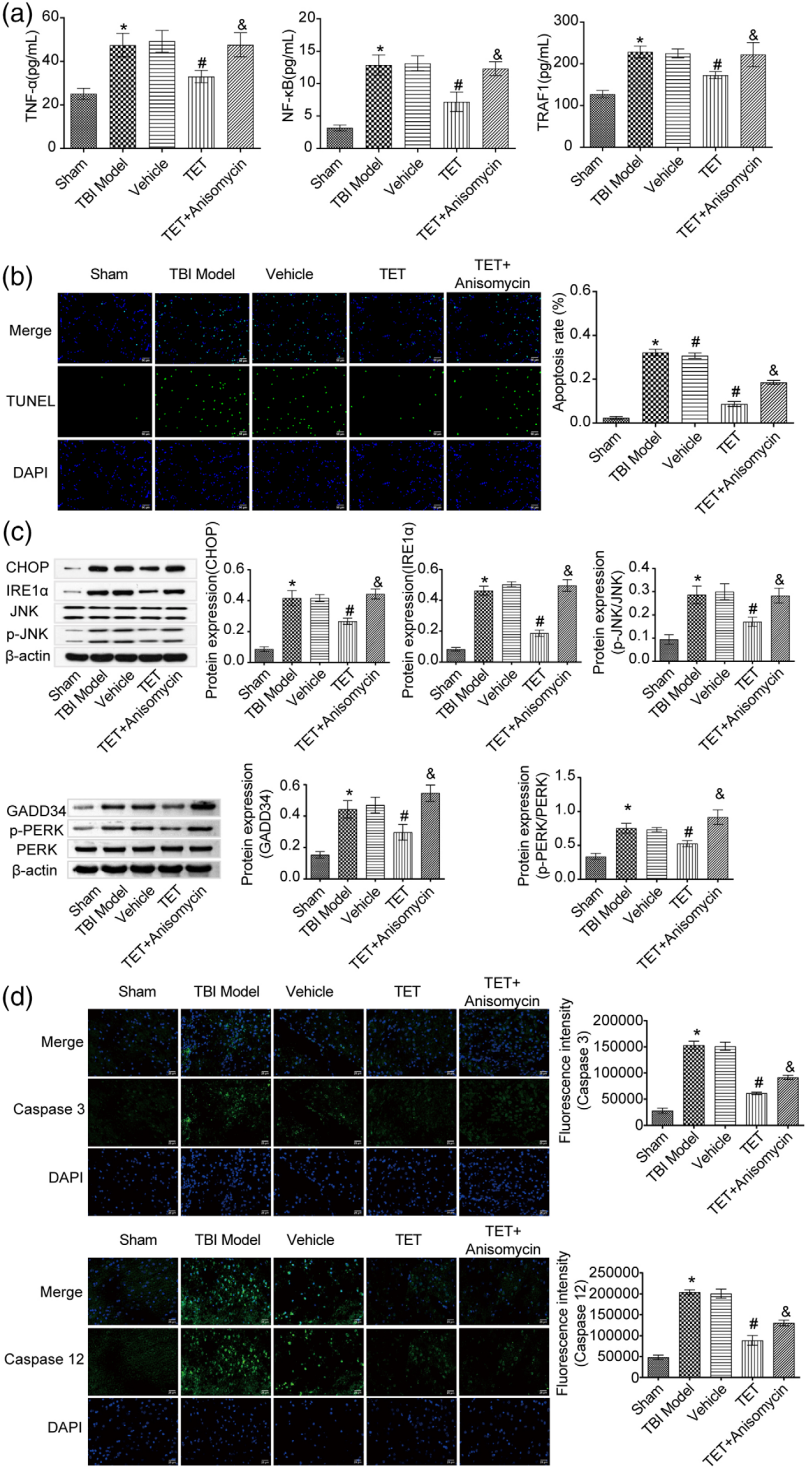
The inflammation and IRE1α/JNK/CHOP signal pathway inhibitory effects of tetrandrine (TET) on traumatic brain injury (TBI) mouse model were reversed by Anisomycin. (a) TNF‐α, NF‐κB, and TRAF1 expression in the hippocampus was evaluated by enzyme‐linked immunosorbent assay (ELISA) assay. (b) TdT‐mediated dUTP nick‐end labeling (TUNEL) staining was used to detect apoptosis. (c) GADD34, PERK, p‐PERK, IRE1α, CHOP, JNK, and p‐JNK expression in the hippocampus was determined by western blot. (d) The expression of Caspase 3 and Caspase 12 was detected by immunofluorescence. *p < .05 versus control group, #*p* < .05 versus vehicle group, &*p* < .05 versus TET group

To determine the effects of TET on apoptosis and the expression of inflammation‐related and pathway‐related proteins in the hippocampal tissue after TBI, ELISA assay, TUNEL assay, and western blot assay were performed on the hippocampal tissues. As shown Figure 5a, TNF‐α, NF‐κB, and TRAF1 in the TBI model were upregulated compared with the sham group, which was downregulated by treating with TET. Interestingly, the expression level of TNF‐α, NF‐κB, and TRAF1 in TET + Anisomycin group was upregulated compared with the TET group. TUNEL results indicated that TET inhibited TBI‐induced apoptosis. Apoptosis was increased in TET + Anisomycin group compared to the TET group (Figure 5b). As shown in Figure 5c, GADD34, p‐PERK, IRE1α, CHOP, and p‐JNK were significantly upregulated in the TBI model group compared with the sham group. TET decreased GADD34, p‐PERK, IRE1α, CHOP, and p‐JNK expression in the hippocampus of TBI mice, which was reversed by the coadministration of Anisomycin. PERK and JNK expression was not statistically significant between groups. Caspase cascades are important effectors in response to CHOP/JNK activation, and markers of endoplasmic reticulum (ER)‐induced apoptosis (Tabas & Ron, 2011; Zhang et al., 2012). Our results suggested that TET diminished Caspase 3 and Caspase 12 cleavage, which was partially revoked by the coadministration of Anisomycin (Figure 5d). The results showed that TET alleviated TBI through the IRE1α/JNK/CHOP signaling pathway.

## 4 DISCUSSION

TBI caused by external mechanical forces to the brain has become a severe global public health problem, which accounts for one‐third of injury‐related deaths in the United States (Taylor et al., 2017). In athletes, sports‐induced TBI damages axons and induces regenerative and degenerative tissue responses in the brain, and repeated concussion may eventually develop into chronic traumatic encephalopathy (Blennow et al., 2012). Neurological influences of TBI encompass both primary injury events that occur at the time of trauma and secondary injury events contributing to subsequent neuronal cell death and tissue damage (Loane & Faden, 2010). These injuries and sequelae bring great inconvenience and threats to daily life of patients (Dixon, 2017). In basic research, TBI model could be constructed on mouse hippocampal neurons by scratch injury to stimulate the TBI‐like biological symptoms (Han et al., 2014). In the present study, the traumatic injury model on hippocampal neurons has been successfully established. The model group showed upregulated NSE expression, apoptosis rate, and inflammation‐related protein expression, including TNF‐α, NF‐κB, and TRAF1. The TBI mice model has been established by surgery, which was described by Teutsch P (Teutsch et al., 2018). In the present study, the results showed that Garcia neuro scores was lower in the TBI model group than in the sham group. Cerebral water content and EB extravasation were increased in the TBI model mice. H&E results showed that the damage of cortex and hippocampus was obvious in the TBI model group. Immunofluorescence results showed that the expression of NSE was increased in the hippocampus of the TBI model group. The therapeutic effects of TET on TBI were evaluated by these models in the present study.

In the pathological processing of secondary TBI, neuro inflammation plays an important role (Corps et al., 2015), which is initiated immediately after the occurrence of TBI and continuously existed in the early stage of craniocerebral injury. Neuro inflammation is reported to be involved in the pathogenesis of secondary TBI and induces delayed neurological impairments, which is mainly resulted from local tissue ischemia and hypoxia, reperfusion injury, free radical injury, overload of intracellular Ca^2+^, and plenty inflammatory cells recruited by necrotic neurons (Clark et al., 2019; McKee & Lukens, 2016). Furthermore, as the aggravation of inflammation, the encephalic microglia and astrocytes are activated, and multiple kinds of inflammatory cells, such as macrophages and neutrophile granulocytes, are infiltrated into the encephalic injury lesions, which induce the release of more inflammatory factors (Karve et al., 2016). In this way, a vicious circle formed to aggravate the severity of secondary TBI. The present vitro study showed that TET significantly downregulated the expression of NSE, TNF‐α, NF‐κB, and TRAF1 in the model group. Severe inflammation was observed in the TBI model mice, which was alleviated by treating with TET. In vivo results were consistent with in vitro results. In addition, TET improved Garcia neuro scores and hippocampal morphological changes, while decreased cerebral water content and EB extravasation in TBI model mice. However, deep investigation on the mechanism underlying the inflammation inhibitory effects of TET will be explored in the nearest future to better understand the therapeutic effects of TET against TBI. Fluorojade staining is used to label degenerating neurons. Fluorojade staining could localize not only the degeneration of neuronal cell bodies but also distal dendrites, axons, and terminals, which could specifically assess the effects of TET on hippocampal neurons with traumatic injury. In Figure 1, the effect of TET on hippocampal neurons with traumatic injury was shown by the detection of NSE by immunofluorescence and the assessment of apoptosis by flow cytometry. Considering the limited funding, we will use Fluorojade staining to further evaluate the effect of TET on hippocampal neurons with traumatic injury in the next project.

Apoptosis of neuron was reported to be involved in the pathogenesis of secondary TBI (X. Chen et al., 2018; Liu et al., 2018). C/EBP homologous protein (CHOP), also known as growth arrest and DNA damage 153, GADD153 and DNA damage‐inducing transcript 3, DDIT3, is a major promoter apoptotic protein that responds to IRE1α and PERK (mainly PERK) signaling pathways (Lerner et al., 2012). CHOP is a transcription factor induced by ERS. CHOP played an important role in cell apoptosis as a pro‐apoptotic transcription factor during cell stress (F. Li et al., 2016). CHOP promoted cell apoptosis by binding and regulating cell death‐related genes such as Bcl‐2, Bim, Bax, and Bad (Ghosh et al., 2012). The previous study has shown that ERS‐induced apoptosis aggravated TBI (G. Sun et al., 2021). TBI could be alleviated by inhibiting the activation of ERS pathway (IRE1, CHOP, etc.) and apoptosis (Deng et al., 2021; Ni et al., 2018). GADD34 and PERK, two of these ERS‐sensing kinases, could affect TBI progression by inducing phosphorylation of the translation initiation factor eIF2α to attenuate new protein translation while triggering the transcriptional program (Dalton et al., 2012; L. Li et al., 2022). C‐Jun N‐terminal kinase (JNK) and its typical target protein c‐Jun were involved in the process of cell apoptosis (Fernandes et al., 2012). Apoptosis factors activated c‐Jun by inducing JNK phosphorylation (Urano et al., 2000). The continuous activation of JNK‐c‐Jun signal induced cell apoptosis by promoting the expression of Bax and inhibiting the expression of Bcl‐2 (Fernandes et al., 2013). In addition, the Caspase cascade is also considered to be an important effector in response to CHOP/JNK activation, such as Caspase 12, Caspase 9, and Caspase 3 (Lakshmanan et al., 2011). Studies have shown that the ERS inhibitor salubrinal reduced the protein expression of CHOP, GADD34, p‐PERK, and p‐eIF2α and the cleavage of Caspase‐3, and inhibited apoptosis, thereby improving blast‐induced TBI (bTBI) (Lucke‐Wold et al., 2017; Logsdon et al., 2014). Salubrinal alleviated bTBI by reducing the expression of p‐JNK, CHOP, NFκB), inducible nitric oxide synthase, TNFα, and interleukin 1 beta (Logsdon et al., 2016). In this study, the results showed that TET inhibited the expression of GADD34 and p‐PERK and the IRE1α/JNK/CHOP signaling pathway activated by the TBI model in vitro and in vivo. Our data indicated that the induced neuron apoptosis by TBI modeling could be suppressed by TET.

Subsequently, Anisomycin, a JNK selective activator, was used to be administered combined with TET to verify whether the effect of TET on neuronal apoptosis and TBI was related to IRE1α/JNK/CHOP signal pathway. The results showed that the downregulated NSE, inhibited apoptosis rate, suppressed inflammation, upregulated GADD34 and p‐PERK, and inactivated IRE1α/JNK/CHOP signal pathway in the TET group were partially offseted by the TET co‐incubation with Anisomycin in vitro. In vivo data were consistent with in vitro data. In addition, TET + Anisomycin decreased the Garcia neuro scores, promoted the morphological changes of the hippocampus, increased the cerebral water content and EB extravasation, and inhibited Caspase 3 and Caspase 12 cleavage. These data indicated that the anti‐TBI effects of TET could be reversed by activating JNK signal pathway. Fluoroscein dextran staining and EB staining could be used to evaluate BBB permeability (Nakano et al., 1997; Yong & Linthicum, 1996). Considering the limited funding, EB staining was used to measure BBB permeability after TBI. In the next project, Fluoroscein dextran staining and EB staining will be used to further evaluate the effect of TET on BBB permeability after TBI and the potential molecular mechanism.

A previous study has shown that propofol protected hippocampal neurons from apoptosis in ischemic brain injury by increasing glial glutamate transporter 1 (GLT‐1) expression and inhibiting N‐methyl‐d‐aspartate (NMDA) receptor activation through the JNK/Akt signaling pathway (Gong et al., 2016). Mechanistically, both the JNK agonist anisomycin and the Akt inhibitor LY294002 significantly blocked the effects of propofol on cell viability and apoptosis of hippocampal neurons in IBI; NMDA reversed the decreased JNK activation and increased Akt activation caused by GLT‐1 overexpression. It has been reported that curcumin significantly improved brain injury and neurological function in rats with middle cerebral artery occlusion by upregulating p‐Akt and p‐mTOR and downregulating LC3‐II/LC3‐I, IL‐1, TLR4, p‐38, and p‐p38, which were reversed by LY294002 (a specific inhibitor of the PI3K/Akt/mTOR pathway) or anisomycin (an activator of TLR4/p38/MAPK) (Huang et al., 2018). Mechanistically, curcumin exerted neuroprotective effects by mediating the PI3K/Akt/mTOR pathway to attenuate autophagy activity while also suppressing an inflammatory reaction by regulating the TLR4/p38/MAPK pathway. In TBI rats, connexin 43 (Cx43) might induce neuronal autophagy by activating P2 × 7R and reduce the expression of GLT‐1 in the hippocampus, promoting cognitive deficit repair (L. Sun et al., 2015). Previous investigations have found that ebselen alleviated TBI by inhibiting the TLR4/p38/MAPK signaling pathway (Wei et al., 2014). Therefore, we reasonably and boldly speculate that the role of anisomycin‐mediated TET in TBI might be closely related to autophagy, GLT‐1, and TLR4/p38/MAPK pathway.

Total flavonoids of Astragalus inhibits microglia‐mediated inflammation in experimental autoimmune encephalomyelitis mice by inactivating the JNK/AKT/NFκB signaling pathway, which was partially abolished by anisomycin (Yang et al., 2021). Salubrinal (ERS inhibitor) provided neuroprotection after TBI by inhibiting the expression of CHOP, autophagy, and apoptosis in neurons, astrocytes, and microglia (Z. F. Wang et al., 2019). In the future work, the underlying molecular mechanism on the effects of TET on the IRE1α/JNK/CHOP signal pathway will be deeply investigated to better understand the pharmacological effects of TET. The study preliminarily clarified that TET might inhibit inflammation and apoptosis through the IRE1α/JNK/CHOP signal pathway, thereby reducing TBI. In the next project, we will combine blocking agents (such as salubrinal) to further the ERS pathway that could mediate the action mechanism of TET in TBI. At the same time, we will use elevated plus maze, open field, or rotarod to evaluate the benefits of the behavior further. We will use the JNK inhibitor (such as SP600125; Tian et al., 2018) to further explore the blocking mechanism of anisomycin‐mediated TET alleviation of TBI.

Taken together, the present study indicated that TET alleviated inflammation and neuron apoptosis in experimental TBI by regulating the IRE1α/JNK/CHOP signal pathway.

## CONFLICT OF INTEREST

The authors declare no conflict of interest.

## Data Availability

The dataset generated during this study is available from the corresponding author upon reasonable request.
